# Insights into Synergistic Effect of Acid on Morphological Control of Vanadium Oxide: Toward High Lithium Storage

**DOI:** 10.1002/advs.202002579

**Published:** 2020-12-03

**Authors:** Yang Zhou, Qiwen Pan, Jing Zhang, Chunmiao Han, Lei Wang, Hui Xu

**Affiliations:** ^1^ Key Laboratory of Functional Inorganic Material Chemistry Chinese Ministry of Education Heilongjiang University 74 Xuefu Road Harbin 150080 P. R. China; ^2^ Energy & Environmental Research Institute of Heilongjiang Province Heilongjiang Academy of Sciences Harbin 150090 P. R. China

**Keywords:** acid radicals, lithium ion batteries, morphology control, protons, synergistic effect

## Abstract

Morphological control is a fundamental challenge of nanomaterial development. Commonly, hierarchical nanostructures cannot be induced by a single driving force, but obtained through balancing multiple driving forces. Here, a feasible strategy is reported based on the synergistic effect of proton and acid anion, leading to the morphological variation of vanadium oxide from nanowire, bundle, to hierarchical nanoflower (HNF). Protons can only induce the formation of nanowire through reducing the pH value ≤ 2. However, acid anions with strong coordination ability, e.g., phosphate radicals, can also participate in morphological regulation at high concentration. Through coordinating with exposed vanadium ions, the enrichment of phosphate radicals at ledge and kink changes the growth directions, giving rise to the advanced structures of bundle and HNF. The lithium ion batteries using HNF as a cathode achieve a 30% improved initial discharge specific capacity of 436.23 mAh g^−1^ at a current density of 0.1 A g^−1^, reaching the theoretical maximum value of vanadium oxide based on insertion/desertion of three lithium ions, in addition to strong cyclic stability at 1 A g^−1^.

## Graphical abstract

Synergistic effect on nanostructure growth of vanadium oxide is studied, taking acid addition as an instant. Protons drive assembly, while acid radicals as phosphate radicals adjusts growth direction through coordinating exposed vanadium ions at ledge and kink. The nanostructure changes from nanowire, bundle, to hierarchical nanoflower, improving the initial discharge specific capacity of a lithium ion battery to the theoretical maximum ≈430 mAh/g.

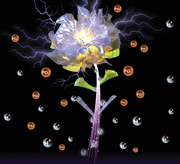

The functionality and performance of nanomaterials are closely associated with the morphology.^[^ ^1^ ^]^ Driving forces of nanomaterial growth are diverse, commonly based on addition of acid, alkali, surfactant, organic skeleton, and so on. Nanomaterials with advanced morphologies for efficient contact with electrolyte are desired as electrodes for lithium ion batteries,^[^ ^2^ ^]^ because of the advantages in volume change during lithium ion storage, agglomeration prevention, active site exposure. For instance, Yu et al. prepared tetragonal PbWO_4_ microcrystals through tuning *p*H value of solution in cetyltrimethylammonium bromide.^[^ ^3^ ^]^ Lou et al. used surfactant and 2‐methylimidazole precursor to prepare triply shelled Co_3_O_4_@Co_3_V_2_O_8_ nanoboxes, which revealed enhanced lithium storage properties.^[^ ^4^ ^]^ These methods demonstrate that the preparation of nanomaterials with hierarchical structures requires synergistic reaction of multiple driving forces. However, the combination of various driving forces from different sources induces the complicated and obscure mechanisms of morphological variation. The insight into the interaction and coordination between different driving forces remains a challenge, but it is fundamentally significant for controllable morphological optimization.

Tuning pH value with acids is a popular method to achieve nanoscale morphology. Vanadium oxide is a kind of famous nanomaterials for Li^+^ storage, either the anode or the cathode,^[^ ^5^ ^]^ However, the morphology of vanadium oxide reported was relatively simple, mostly nanorod^[^ ^6^ ^]^ or nanoparticle.^[^ ^7^ ^]^ The morphology can be modified to nanowire, through using acid to tune pH value in the range of 2–7.^[^ ^8^ ^]^ Nonetheless, for lithium ion battery application, hierarchical nanostructures are desired. It is noted that in PH value range of 2–7, proton is the main driving force to control the self‐assembly of vanadium oxide particles and modulate material morphology. Therefore, no vanadium oxide with advanced nanoscale morphology is constructed just through acid addition, inducing the insufficiently exploited lithium storage potential. It can be noted that even at pH = 2, acid concentration is still as low as 0.01 M in the case of a first order dissociation. Until present, no attempt was made to further increase acid concentration. However, when pH approaching to 1, acid anion as the counterpart of proton becomes considerable quantity. In this case, disassociated acid provides two driving forces to influence the growth of vanadium oxide and regulate morphology. While, as integrity, proton‐acid anion interaction is straightforward and less influential on investigating combined effect of acid.

In this contribution, through gradually decreasing pH value from 2 to 1, we found vanadium oxide nanowire assembles into bundle and then hierarchical nanoflower (HNF). However, this growth process is strongly dependent on the existence of acid anions with strong coordination ability, especially phosphate radical. Otherwise, only nanowire can be obtained, despite pH = 1. The results of electron microscopy, X‐ray diffraction (XRD), and Raman spectra suggest through coordination with exposed vanadium ions, phosphate radicals accumulate at ledge and kink and restrain the original growth directions, leading to the formation of 3D nanostructures. The specific surface area and active V^4+^ density are directly proportional to structural dimensionalities. Consequently, HNF as electrode of lithium ion batteries realized a theoretically maximum initial discharge specific capacity of 436.23 mAh g^−1^, which is improved by 30% in contrast to nanowire. This work demonstrates the synergistic effect of acid in morphological adjustment, based on proton‐facilitated assembling and acid anion‐controlled growth direction, providing an insight into the coordination mode of driving forces in constructing hierarchical nanostructures.

We systematically investigated the influence of diverse acids on nanostructure growth in a large pH range of 0.5–3.0. The vanadium oxide nanomaterials were prepared by hydrothermal approach, respectively incorporating sulfuric acid, nitric acid, hydrochloric acid, phosphoric acid, formic acid and acetic acid. As long as pH exceeding a critical value, addition of sulfuric acid (pH < 1.8), nitric acid (pH < 2.3), formic acid (2 < pH < 3) and acetic acid (1.5 < pH < 1.8) always induce the formation of nanowire, which is consistent with previous report (Figure S1, Supporting Information).^[^ ^9^ ^]^ In contrast, when adopting phosphoric acid, nanowire is formed in a pH range of 1.3–3.0, which achieves the highest crystallinity and yield at pH = 1.8, a high pH (**Figure** **1**a). Decreasing pH to 1.3 leads to the formation of bundle structure based on nanowires (Figure 1b). More significantly, at pH = 1, the material nanostructure further evolves into three‐dimensional HNF as shown in Figure 1c. The situation of hydrochloric acid is similar to that of phosphoric acid (Figure S2, Supporting Information). In cases of sulfuric acid, nitric acid, formic acid and acetic acid, once formed, the nanowire structure is independent to acid concentration variation, which suggests proton is the single driving force. The formation of advanced nanostructures at high phosphoric acid concentration reflects a nonlinear growth mode based on a balance between multiple driving forces.

**Figure 1:**
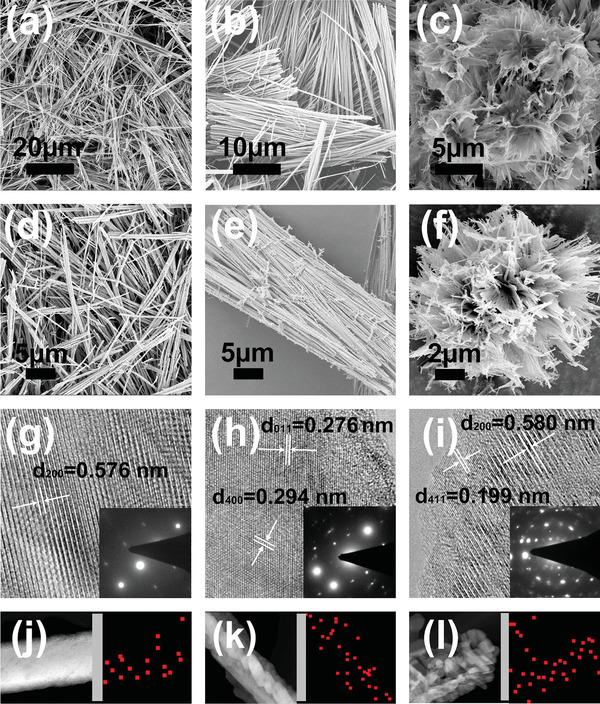
Structure and composition of nanowire, bundle, and HNF prepared with phosphoric acid. SEM images of a) nanowire, b) bundle, and c) HNF as prepared; SEM of d) nanowire, e) bundle, and f) HNF annealed at 400 °C, and g–i) corresponding HRTEM images. The inset shows SAED pattern; elemental mapping images of phosphorus in j) nanowire, k) bundle, and l) HNF after annealing.

The optimal annealing temperature was chosen as 400 °C, according to thermo‐gravimetric analysis (TGA) (Figure S3, Supporting Information). Scanning electron microscopy (SEM) shows that after annealing at 400 °C in air, the materials retain the nanostructures, in addition to the further crystalized primary grains (Figure 1d–f and Figure S4, Supporting Information). For nanomaterials prepared with phosphoric acid, representative lattices of different grains can be observed from high‐resolution transmission electron microscopy (HRTEM) (Figure 1g–i). Selected area electron diffraction (SAED) patterns indicate the crystal lattice of each grain in nanowire is neatly arranged, due to the ordered growth along crystal plane. In contrast, besides the growth crystal plane similar to nanowire, bundle reveals another growth direction. The lattice growth in HNF is further differentiated, resulting in multiple crystal planes. Notably, according to elemental mapping, there are considerable phosphorus elements existing in the materials (Figure 1j–l). The phosphorus amount is proportional to the structural complexity, indicating the incorporation of phosphate radical in the formation of hierarchical structure.

In comparison to the standard spectrum (Figure S5, Supporting Information), X‐ray diffraction (XRD) pattern of nanowire matches well with that of an orthorhombic V_2_O_5_ phase (**Figure** **2**a). XRD data of HNF are in good agreement with the H_0.39_V_2_O_5_. 2*θ* peak positions of nanowire, bundle, and HNF are approximate, but their peak intensities are different (Figure 2b). 2*θ* of nanowire (301) is in accord to those of bundle and HNF (400), reflecting a crystal plane evolution. Furthermore, the ratio of (301) for nanowire and (400) for bundle and HNF to their (110) decreases from 10:7 to 7:10, due to the enhanced (110) intensities of bundle and HNF. Similarly, the intensities of the (200) and (101) peaks are also increased for bundle and HNF, which is in accord to SAED patterns (Figure 1g,i). The XRD peak intensities of HNF are mostly larger than those of nanowire, reflecting the improved crystallization of every crystal plane in HNF. From nanowire, bundle to HNF, the material growths involve in more and more crystal planes, inducing the increase in the dimensionality of nanostructure. In high supersaturation acid system of vanadate, nanostructure growth follows a Kossel Stranski Volmer (KSV) model.^[^ ^10^ ^]^ Vanadium oxide assemblies along (301) and (001) crystal planes under the control of proton. High phosphoric acid concentration provides sufficient phosphate radicals, which are enriched at ledge and kink through direct interactions to vanadium ions. It hinders the growth of (301) and (001) crystal planes, and in turn facilitates the growth along (400) and (110) crystal planes. This process is in accord to SAED and elemental mapping results. According to (400) crystal plane (Figures 1h and 2b), bundle with less than 0.39 hydrogen ions corresponds to a transition state from V_2_O_5_ to H_0.39_V_2_O_5_. Therefore, despite the evolution from (301) to (400) crystal planes, phosphate radical amount is not enough to simultaneously support the growth along (110) crystal plane of bundle.

**Figure 2:**
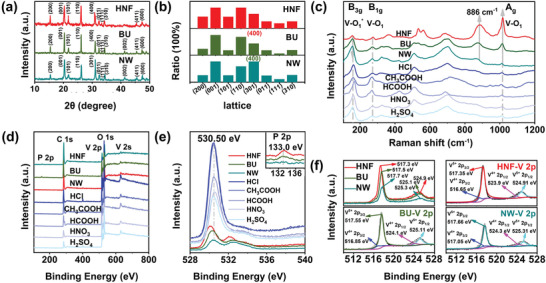
Interactions between acid and vanadium oxide nanomaterials. a) XRD patterns of nanowire (NW), bundle (BU), and HNF prepared with phosphoric acid; b) peak intensity comparison between selected crystal planes of nanowire, bundle, and HNF; c) Raman spectra of nanowire, bundle, and HNF prepared with phosphoric acid and nanowires prepared with other acids. Except phosphoric acid, other acids are marked on the corresponding curves; d) XPS wide spectra and magnified e) P 2p region (inset) and O 1s region of nanowire, bundle, and HNF prepared with phosphoric acid; f) V 2p region and multipeak fitting of nanowire, bundle, and HNF prepared with phosphoric acid.

The growth kinetics of HNF was investigated through monitoring the morphological variation at different reaction times (Figure S6, Supporting Information). It is showed that under phosphoric acid concentration of 28 mmoL (pH = 1), bulky precipitates are gradually fragmented within the initial 20 min, and exfoliated into nanoparticles with diverse aggregation degrees in the next 1 h. Nanoparticles assemble into nanowires along (301) and (001) crystal planes in 2–5 h. Then, longitudinal growth of nanowires forms bundles. The third dimensional growth along (400) and (110) crystal planes finally generates HNFs. The growth process of HNF has a step‐by‐step feature. It means the formation of HNF is dependent on the growth direction change from one dimension to three dimensions. The ledges and kinks on the surfaces of nanowires and bundles should be formed first to provide the sites for enrichment of phosphate radicals. Then, differential distribution of phosphate radicals changes growth direction.

The interactions between acid anions and nanomaterials are further determined with Raman spectra (Figure 2c). Nanowires prepared with different acids reveal the similar spectra. However, the use of phosphoric acid gives rise to a characteristic absorption peak at 886 cm^−1^, corresponding to the *γ*_1_(*A*_1_) stretching vibration of P—O (1.60 Å).^[^ ^11^ ^]^ Bundle and HNF also display this absorption peak with gradually increased intensities. Moreover, the absorption of the characteristic O—V—O bending vibration (B_3g_) at 146 cm^−1^ is remarkably weakened in the spectra of bundle and HNF, accompanied by enhanced V=O stretching vibration peak at 1011 cm^−1^.^[^ ^12^ ^]^ These results indicate the interaction between phosphate radical and vanadium oxide is based on P—O→V coordination, which impedes O—V—O bending vibration, but enhances double‐bond character of V=O. The ledges and kinks provide sufficient exposed vanadium ions, leading to coordination induced enrichment of phosphate radicals. The two characteristic peaks of V_2_O_5_ remain the same in other samples, since coordination abilities of the other anions are weaker than phosphate radical anion.

X‐ray photoelectron spectroscopy (XPS) gives another evidence of coordination between phosphate radical and vanadium oxide (Figure 2d). P 2p peaked at 133.00 eV indicates the existence of P‐O,^[^ ^13^ ^]^ whose intensity is remarkably increased from nanowire, bundle to HNF (inset of Figure 2e). The synchronous interactions to O in P=O→V reduces electron cloud density of O, leading to the stronger binding energy of O nucleus to 1s electron. Therefore, compared to nanowires prepared with other acids, the use of phosphoric acid induces O 1s peak shifted from 530.50 to 532.05–532.40 eV (Figure 2e).^[^ ^14^ ^]^

In turn, P=O→V coordination increases electron cloud density of V, enhancing electron shielding effect on nucleus binding. Thus, V 2p peaks shift to the low energy region, in which V 2p 2/3 and 2p 1/2 respectively shift from 517.70 and 525.30 eV to 517.30 and 524.90 eV (Figure 2f). Simultaneously, V^4+^ excitation peaks are remarkably enhanced, due to the increased oxygen vacancies at ledges and kinks in bundle and HNF,^[^ ^15^ ^]^ which is in accord to the result of electron paramagnetic resonance (EPR) spectroscopy (Figure S7, Supporting Information).^[^ ^16^ ^]^

To further demonstrate the indispensable of phosphate radical in HNF formation, a control experiment was design by using nitric acid as proton source and ammonium dihydrogen phosphate as phosphate source. Without phosphate, addition of nitric acid can only induce the formation of nanowire. It means only with pH value ≥ 1, the morphology remains the nanowires without phosphate radical (Figure S1e, Supporting Information). In this sense, proton induces nanowires grow longer and longer. However, the combined use of nitric acid and phosphate radical gives rise to desired HNF morphology, identical to the structure obtained by using phosphoric acid (Figure S8, Supporting Information). It is confirmed that the existence of phosphate radical can change the growth direction of nanowires, resulting in the formation of nanoflower structures.

It means sufficient proton and phosphate radical are two necessary and sufficient conditions for achieving three‐dimensional nanostructures.

These results indicate the growth process of HNF contains three main steps: i) proton‐facilitated vanadium oxide assembly, inducing the linear growth along (301) and (001) crystal planes; ii) formation of ledges and kinks, providing exposed vanadium ions; iii) enrichment of phosphate radical at ledges and kinks through coordination to exposed vanadium ions. The coordination restraint in turn changes the growth direction along (400) and (110) crystal planes, giving rise to hierarchical nanostructure (**Scheme** **1**). As two aspects of synergistic effect, proton is the first driving force for nanoparticle assembly, and the coordination effect of phosphate radical is the second driving force for adjusting growth directions. The difference of the acids in morphological control is mainly embodied in the coordination ability of their acid anions.

**Scheme 1:**
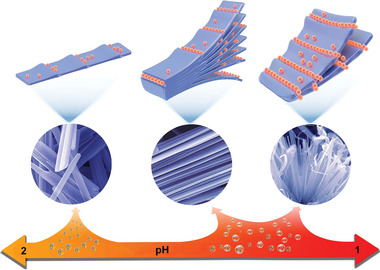
The growth mechanism of vanadium oxide nanomaterials prepared at different concentrations of phosphoric acid. Proton first induces vanadium oxide assembly, accompanied by formation of ledges and kinks on (301) and (001) crystal planes. High‐concentration phosphoric acid provides sufficient phosphate radicals (orange balls) to coordinate with exposed vanadium ions at ledges and kinks. The coordination changes growth direction, leading to formation of hierarchical nanostructure.

After calcinated at 400 °C to improve crystallinity, specific surface areas of nanowire, bundle and HNF materials are respectively 10.42, 23.01, and 32.69 m^2^ g^−1^, which are in direct proportion to the morphological dimensionality (Figure S9, Supporting Information). The curves of nanowire and bundle are type‐III isotherm, indicating weak forces between the powder and nitrogen. In contrast, the curve of HNF is a type‐IV adsorption loop, which means spaces between adjacent HNFs also contribute to nitrogen absorption.^[^ ^17^ ^]^ 3D hierarchical structure of HNF is beneficial to Li ion insertion/extraction processes, therefore effectively mitigating volume change.^[^ ^18^ ^]^

Nanowire, bundle and HNF were used as cathodes to assemble lithium ion battery. Cyclic voltammetry (CV) analysis was performed at a scan rate of 0.1 mV s^−1^ in the range of 1.5–4.0 V (vs Li/Li^+^). HNF shows clear phase transition *α* → *ε* → *δ* → *γ* → *ω* phase during redox process (**Figure** **3**a), corresponding to the typical phase transitions of V_2_O_5_ materials,^[^ ^19^ ^]^ which reflects the similar Li^+^ diffusion pathway in HNF during intercalation and deintercalation of lithium ion.^[^ ^20^ ^]^ For HNF, reaction process of lithium intercalation and deintercalation is based on

(1) $$\begin{array}{c} H_{0.39}V_{2}O_{5}+x\mathrm{Li}+xe^{-\;}↔Li_{x}H_{0.39}V_{2}O_{5}\;\left(0\leq x\leq 3\right) \end{array}$$

in which *x* is lithium number. At the first reduction peak at 3.31 V, *α*‐phase is formed when 0 ≤ *x* ≤ 0.10, corresponding to slightly higher discharge specific capacity of ≈47 mAh g^−1^ for bundle and HNF than nanowire (Figure 3b). When 0.1 < *x* ≤ 0.35, *ε*‐phase is generated at 3.08 V under a relatively strong current. The contribution of specific capacity is about 75 mAh g^−1^. *δ*‐phase is then formed in the range of 0.35 < *x* ≤ 1, giving rise to two CV peaks at 2.32 V and 2.21 V, respectively, and a specific capacity of 25 mAh g^−1^. These stages complete intercalation of the first Li^+^ ion. In the ranges of 1 < *x* ≤ 2 and 2 < *x* ≤ 3, *γ*‐ and *ω*‐phases are formed at the reduction peaks of 2.01 and 1.78 V, respectively. In the subsequent anodic scans, the oxidation peaks at 2.77 and 3.47 V are respectively is attributed to the deintercalation of lithium ion and the phase transformation from *ω*‐ to *δ*‐phases, and then to *ε*‐phase.

**Figure 3:**
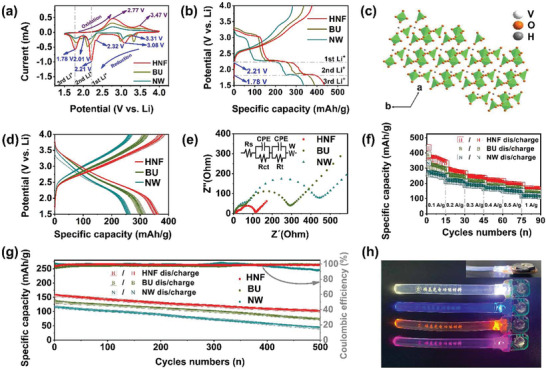
Lithium storage performance. a) CV curves of the three materials with different morphologies at a voltage range of 1.5–4 V (vs Li^+^/Li) and the scan rate of 0.1 mV s^−1^; b) galvanostatic first cycle discharge/charge voltage profiles of nanowire (NW), bundle (BU), and hierarchical nanoflower (HNF) at a current density of 0.1 A g^−1^; c) crystal structures of HNF; d) galvanostatic from the second cycle to the 11th cycle discharge/charge voltage profiles of the three materials with different morphologies at a current density of 0.1 A g^−1^; e) Nyquist plots and equivalent circuits of the three materials with different morphologies; f) rate capability of the three materials with different morphologies at multiple current densities; g) long‐term cycle stability of the three materials with different morphologies at a current density of 1 A g^−1^; h) photographs of LED light powered by the HNF‐400 battery.

By comparing CV and discharge curves of Li^+^ intercalation, HNF realizes the specific capacity of 436.23 mAh g^−1^, reaching to the theoretically maximum specific capacity based on three Li^+^ ions intercalation (Table S1, Supporting Information). This value is 12% and 30% larger than those of bundle and nanowire, respectively. Moreover, the HNF exhibits a specific capacity much higher than the reported vanadium oxide‐based materials with diverse morphologies (Table S2, Supporting Information). As shown by XRD and EPR results, more exposed V^4+^ in hierarchical structure and inside V^4+^ due to H^+^ insertion in crystal lattices (Figure 3c) give rise to the improved specific capacity of HNF. No cathodic peaks can be observed after the second cycle of HNF, due to the formation of amorphous vanadium oxide (Figure 3d). Nevertheless, HNF retains a stable specific capacity of 350–370 mAh g^−1^.

According to electrochemical impedance spectra, HNF exhibits a smaller charge transfer resistance (*R*_ct_) value of 205.2 Ω than bundle (312.1 Ω) and nanowire (404.5 Ω), owing to more exposed V^4+^ active sites in HNF (Figure 3e and Table S2, Supporting Information).^[^ ^2^, ^21^ ^]^ Even at the current density of 1 A g^−1^, the specific capacity of HNF can reach 150 mAh g^−1^, displaying the best rate performance (Figure 3f). Furthermore, cycling performance is another important evaluation criterion of battery performance. Figure S11 (Supporting Information) showed a voltage fade with the discharge voltage decreased by 7.5% from 3.5868 V to 3.3190 V after 500 cycles, primarily due to irreversible phase transition of layered oxides cathodes.^[^ ^22^ ^]^ Nevertheless, the specific capacity of HNF retains 102 mAh g^−1^ after 500 cycles at the current density of 1 A g^−1^, corresponding to a 99% coulombic efficiency, which manifests the excellent cycle stability (Figure 3g). The battery based on HNF can be used to light LED lamp (Figure 3h), indicating the potential in the portable power source applications.

In summary, the synergistic effect of acid on morphological control is investigated on the basis of different vanadium oxide nanostructures. In contrast to other acids, phosphoric acid induces the morphological evolution from nanowire, bundle to HNF through tuning *p*H value. It is showed that the formation of hierarchical nanostructure is based on synergistic effect of proton and phosphate radical. Proton is the first driving force inducing linear growth through vanadium oxide assembly. The coordination effect of phosphate radical is the second driving force for adjusting growth directions, through coordinating with exposed vanadium ions at ledge and kink. The hierarchical nanostructure gives rise to more V^4+^ active sites of HNF. Therefore, HNF endowed its lithium ion batteries with a 30% improved initial discharge specific capacity of 436.23 mAh g^−1^, reaching the theoretical maximum value of vanadium oxide, and the excellent cyclic stability. These findings give insights into the synergistic effect in nanomaterial morphology control, which are crucial to material design and performance improvement.

## Conflict of Interest

The authors declare no conflict of interest.

## Supporting information

Supporting Information
