# Supplementary material for: Insights into Synergistic Effect of Acid on Morphological Control of Vanadium Oxide: Toward High Lithium Storage
Source: Adv Sci (Weinh). 2020 Dec 3;8(2):2002579. doi: 10.1002/advs.202002579 (PMC7816703; doi:10.1002/advs.202002579)
Supplement: Supplementary file 1 — Supporting Information [file ADVS-8-2002579-s001.pdf]

© 2020 Wiley-VCH GmbH

ADVANCED SCIENCE
Open Access

Supporting Information

for *Adv. Sci.*, DOI: 10.1002/advs.202002579

Insights into Synergistic Effect of acid on Morphological Control of Vanadium Oxide: Towards High Lithium Storage

Yang Zhou,[a][b] Qiwen Pan,[a] Jing Zhang,[a] Chunmiao Han,[a] Lei Wang[a] and Hui Xu*[a]*

Supporting Information

Insights into synergistic effect of acid on morphological control of vanadium oxide: towards high lithium storage

Yang Zhou,[a][b] Qiwen Pan,[a] Jing Zhang,[a] Chunmiao Han,[a] Lei Wang*[a] and Hui Xu*[a]

[a] Key Laboratory of Functional Inorganic Material Chemistry, Chinese Ministry of Education, Heilongjiang University, 74 Xuefu Road, Harbin 150080, P. R. China.

[b] Energy & Environmental Research Institute of Heilongjiang Province, Heilongjiang Academy of Sciences, Harbin 150090, P. R. China.

* E-mail address: wanglei0525@hlju.edu.cn (LW); hxu@hlju.edu.cn (HX)

Experimental Section

1. *Synthesis of the products*

Chemical Materials: The ammonium metavanadate (NH_4VO_3) was purchased from aladdin. The sulfuric acid (H_2SO_4), nitric acid (H_3NO_4), hydrochloric acid (HCl), phosphoric acid (H_3PO_4), formic acid (HCOOH), acetic acid (CH_3COOH)，ammonium dihydrogen phosphate（$NH_4H_2PO_4$） and ethanol(C_2H_5OH) were purchased from Kermel Chemical Reagent Co., Ltd (Tianjin, China). All regents were used directly without further purification. The deionized water is the solvent in all experiments.

Synthesis of HNF, BU and NW: In a typical synthesis, 0.06g NH_4VO_3 was dissolved in 40 mL deionized water with gentle stirring. Phosphoric acids were added into the aqueous of ammonium metavanadate in order to adjust the pH of the solution within a certain range. Then, the mixture was transferred into a Teflon-lined autoclave (50 mL) and hydrothermally reacted at 160 ^{o}C for 12 h. The resultant product was collected by centrifugation, and rinsed thoroughly with distilled water and ethanol. The nanomaterials were obtained and then dried at 60 ^{o}C for 12h in an electric thermostatic drying oven. When the mixture is pH 1, the hierarchical nanoflowers sample was named HNF. When the mixture is pH 1.3, the buddle sample was named BU. When the mixture is pH 1.8, the nanowire sample was named NW.

Synthesis of compared samples: The basic experimental process was according to the synthesis of HNF. Hydrochloric acid (pH=0.5; pH=0.66; 0.66<pH<0.95) can adjust the sample to three morphologies. However, sulfuric acid (pH<1.8), nitric acid (pH<2.3), formic acid (2< pH<3), acetic acid (1.5< pH<1.8) all can be prepared into nanowire. Control the system of pH=1 by a certain concentration of nitric acid. Then 0.06 g $NH_4H_2PO_4$ was added and stirred for 30 mins to form transparent solution. The obtained solution was then transferred to Teflon-lined autoclave.

Annealing of samples: In order to study the lithium electrical properties of three representative morphologies, they were annealed at 350 ^{o}C、400 ^{o}C and 450 ^{o}C, for 2h at the rate of 2 ^{o}C /min in air atmosphere, respectively. The HNF sample calcined at 350 ^{o}C is named HNF-350. So on and so forth, we can get HNF-400, HNF-450, BU-350, BU-400, BU-450, NW-350, NW-400 and NW-450.

2. *Characterizations*

SEM characterizations were taken on Hitachi S-4800 field emission scanning electron microscope. HRTEM images were obtained on Titan G2 60-300(AC-TEM), Super X high resolution transmission electron microscopy, with the EDS mapping. XRD characterizations

were taken on the instrument of Bruker D8, LynxEye with the test cases Cu Kα (λ=1.5406Å) and the scanning speed of 8º min^{-1}. Raman tests were conducted at an excitation wavelength of 532 nm using a Invia010410 confocal Raman microscope. XPS was tested on VG ESCALABMK Ⅱ. TGA were detected on TA Q600 at the rate of temperature 5℃/min. BET were analyzed on TriStar Ⅱ 3020 automatic analyzer with the liquid nitrogen temperature 77 K. The continuous-wave (CW) X-band EPR spectra were recorded under 2 K on Bruker A300.

3. Assembly and test of Li-ion batteries

Preparation of electrode: The electrode was prepared by scraping coating. N-methylpyrrolidione (NMP) regulated the viscosity of the active substance (vanadium oxide), binder (PVDF) and conductive carbon (mass ratio of 8:1:1), the suitable slurry was uniformly coated on collector (aluminum foil). The coated aluminum foils were dried in an oven of 80 ^{o}C, 2h, then in a vacuum dryer 110 ^{o}C for 12 h. The samples were cut into wafers with a diameter of 14 mm and transferred into a glove box with argon atmosphere (Etelux), which can be used as a Lithium ion battery positive electrode for battery assemble. The mass of active substance is in the range of 1.5-2.0 mg.

Assemble of Li-ion cell: The prepared electrodes were assembled in a glove box filled with argon gas, and the environment in the glove box maintains water and oxygen content under 1 ppm. When assembling the battery, the prepared electrode was used as the working one, the metal lithium sheet was used as the opposite one, and the diaphragm was Celgard 2400 microporous polypropylene film. The electrolyte was $LiPF_6$ solution of 1 mol/L (PC:EC:DMC=1:1:1). Assemble of CR2025 battery is stacked and sealed in the order of negative, shim, metal lithium, diaphragm (with 4-6 drops of electrolyte), electrode, shim, spring sheet and positive. Then the batteries at room temperature for several hours to be tested.

Test of Li-ion batteries: The electrochemical cyclic voltammetry (CV) and electrochemical impedance spectroscopy (EIS) were carried on the Autolab workstation produced by wantong, Switzerland. The CV test conditions were voltage range of 1.5-4 V, the scan rate of 0.1 mV/s. The EIS test conditions were as follows: voltage range 0.02-3 V, scan rate 0.2 mV/s, AC voltage 5 mV, frequency range 10-100 kHz.

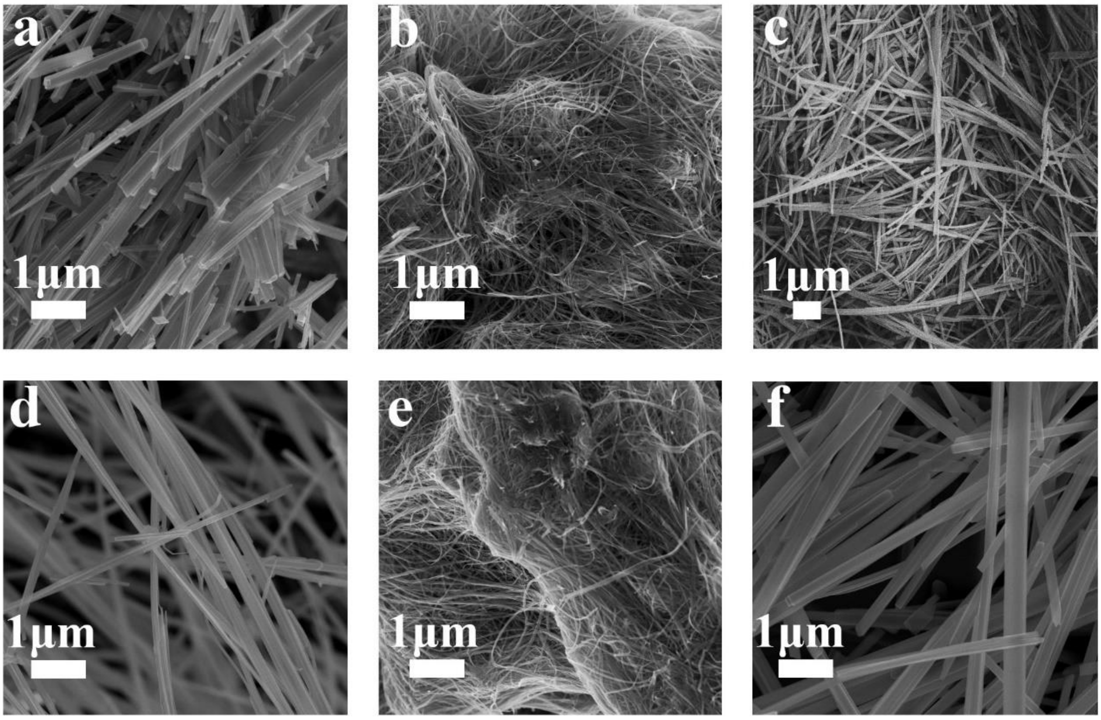

Figure S1. SEM images of the nanowires prepared by sulfuric acid (**a**: pH = 1.8, **b**: pH = 1.0), formic acid (**c**: pH = 2.2), nitric acid (**d**: pH = 2.0, **e**: pH = 1.0), acetic acid (**f**: pH = 3.5).

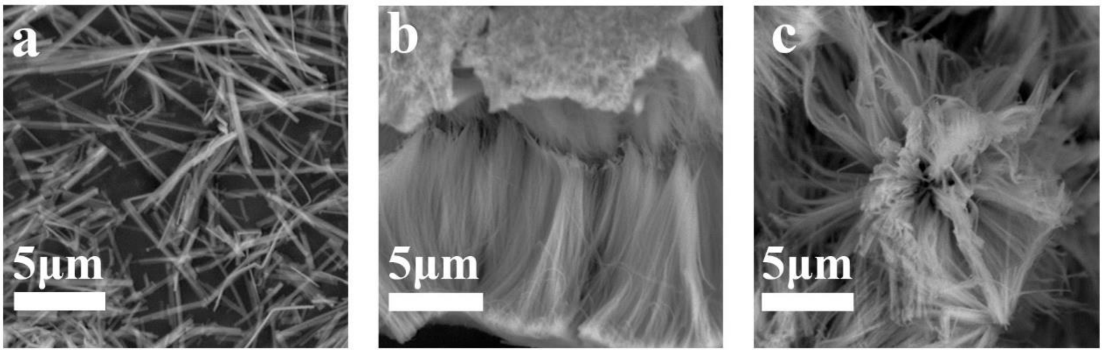

Figure S2. SEM images of nanowire (**a**: pH = 0.95), bundle (**b**: pH = 0.66) and nanoflower (**c**: pH = 0.5) with different morphologies prepared with hydrochloric acid.

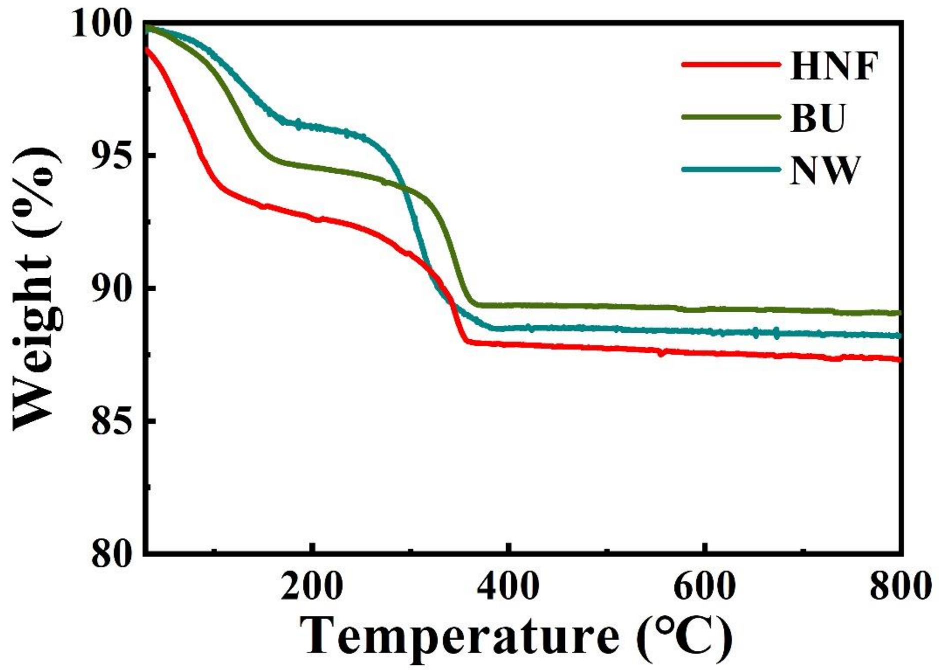

Figure S3. Thermogravimetric analysis (TGA) of nanowire (NW), bundle (BU) and hierarchical nanoflower (HNF) prepared with phosphoric acid.

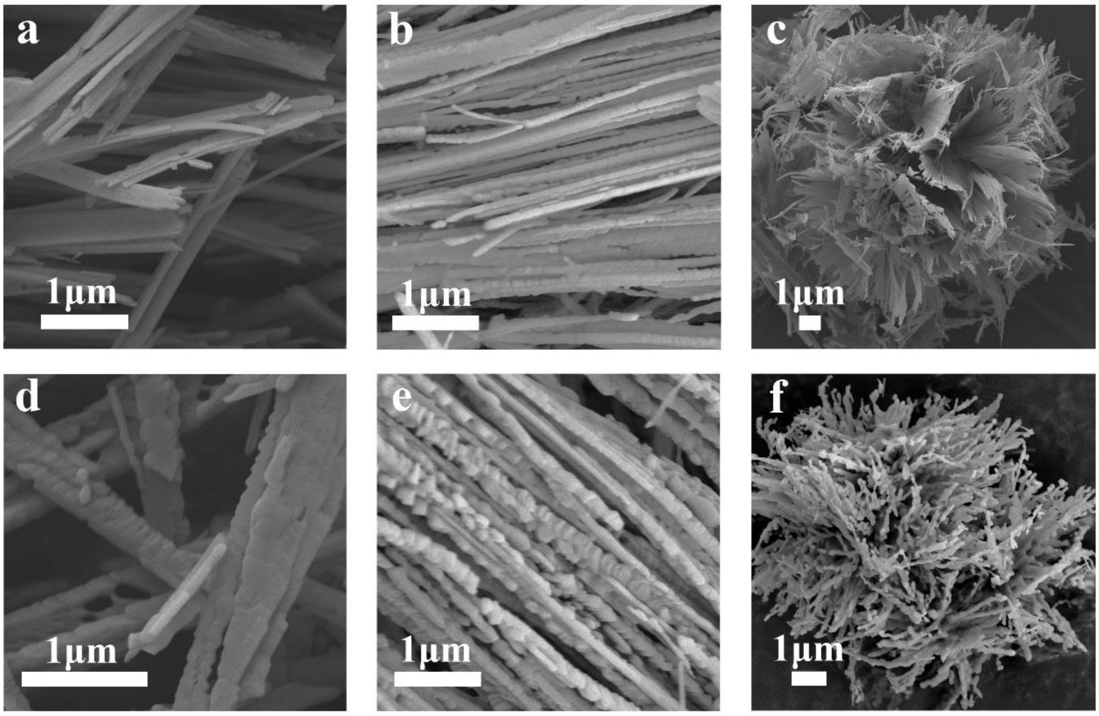

Figure S4. SEM images of nanowire (**a**) annealed at 350 ^{o}C, bundle (**b**) annealed at 350 ^{o}C, and nanoflower (**c**) annealed at 350 ^{o}C; SEM images of nanowire (**d**) annealed at 450 ^{o}C, bundle (**e**) annealed at 450 ^{o}C, and nanoflower (**f**) annealed at 450 ^{o}C. The materials were prepared with phosphoric acid.

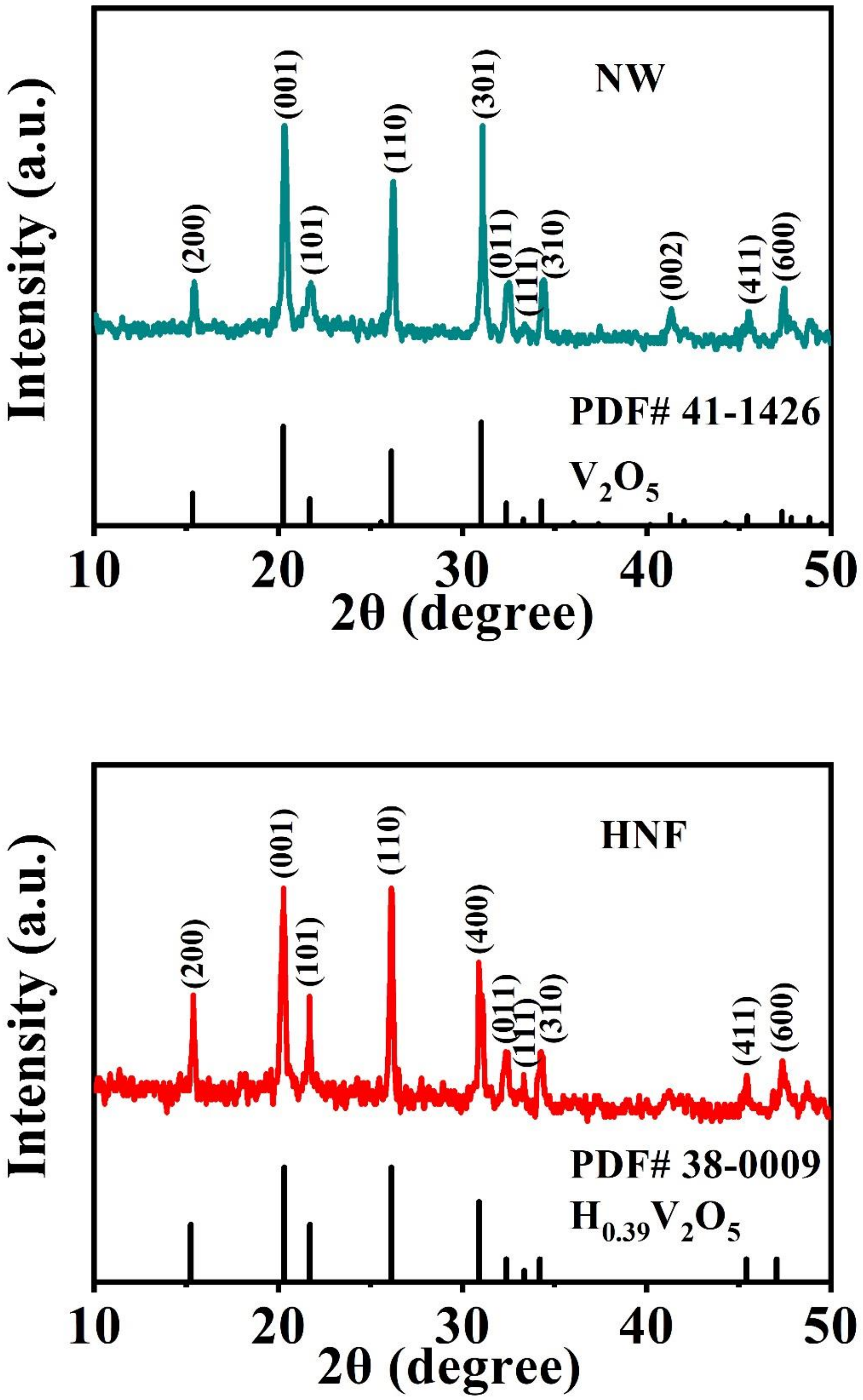

Figure S5. a) XRD pattern of nanowire (NW) and V_2O_5 card (PDF#41-1426); **b)** XRD pattern of hierarchical nanoflower (HNF) and $H_{0.39}V_2O_5$ card (PDF#38-0009). The materials were prepared with phosphoric acid.

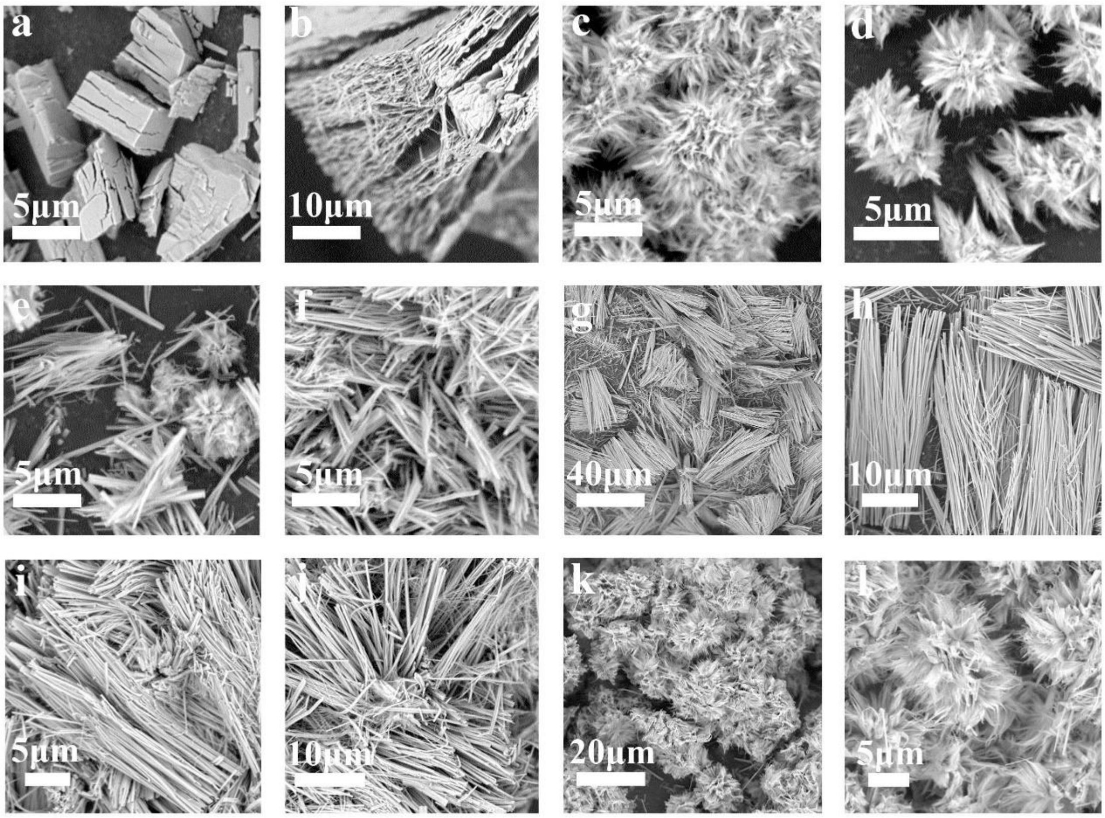

Figure S6. SEM images of hierarchical nanoflower (HNF) prepared with phosphoric acid at different reaction times of hydrothermal process: 20 min (**a**, **b**), 1 h (**c**, **d**), 2 h (**e**, **f**), 5 h (**g**, **h**), 7 h (**i**, **j**), 12 h (**k**, **l**).

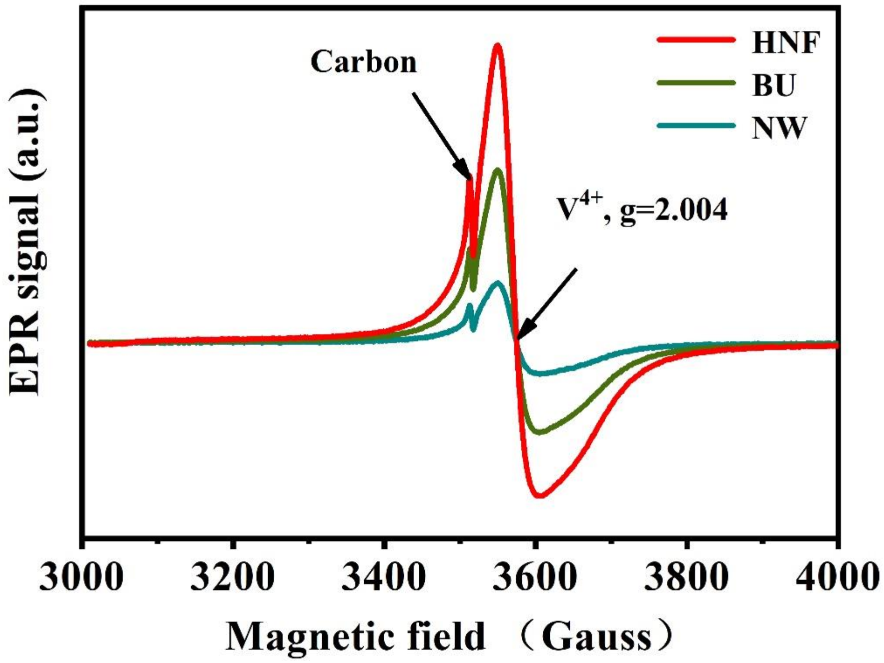

Figure S7. EPR spectra of nanowire (NW), bundle (BU) and hierarchical nanoflower (HNF) prepared with phosphoric acid.

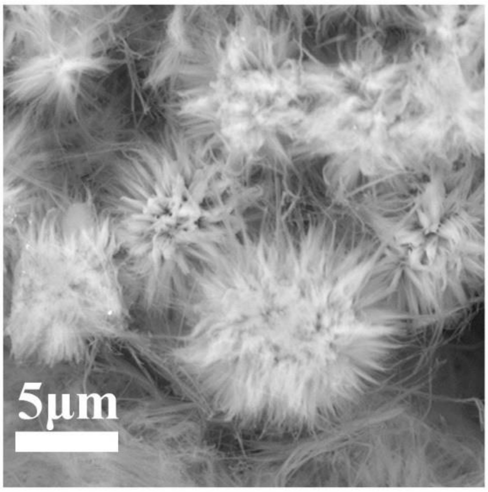

Figure S8. SEM image of hierarchical nanoflower (HNF) prepared by co-addition of nitric acid and ammonium dihydrogen phosphate.

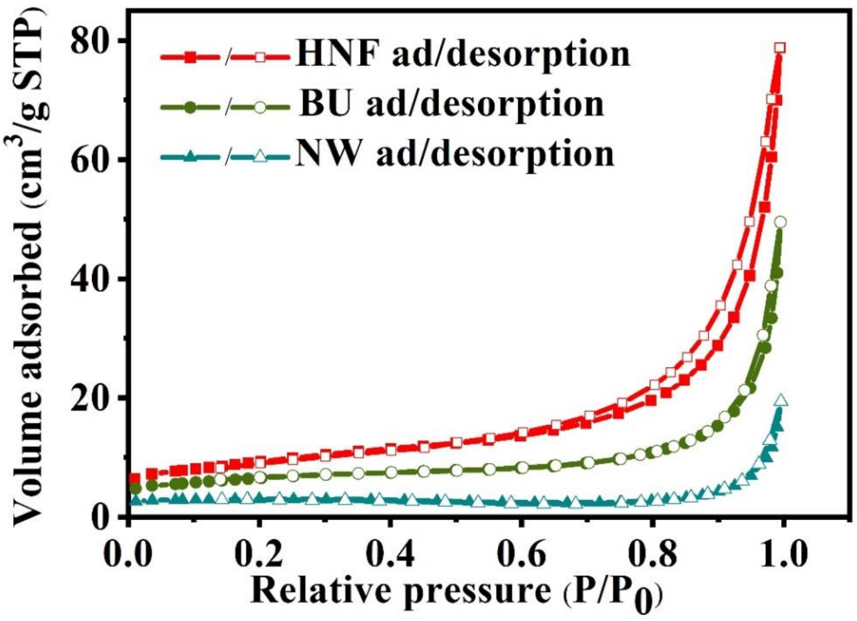

Figure S9. Nitrogen adsorption-desorption isotherms of nanowire (NW), bundle (BU) and hierarchical nanoflower (HNF) prepared with phosphoric acid.

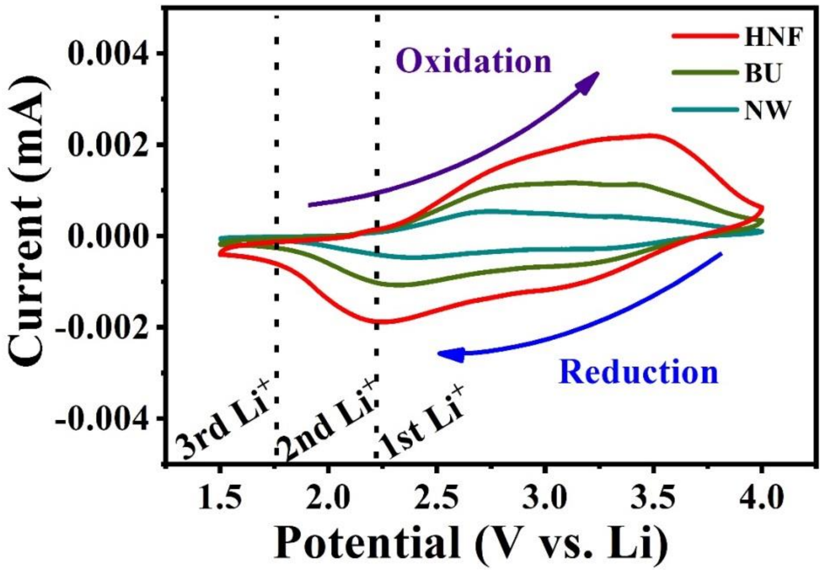

Figure S10. The second scan interval of the CV curves of nanowire (NW), bundle (BU) and hierarchical nanoflower (HNF) prepared with phosphoric acid, at the scan rate of 0.1 mV/s, and from the 1.5-4 V (vs. Li^+/Li).

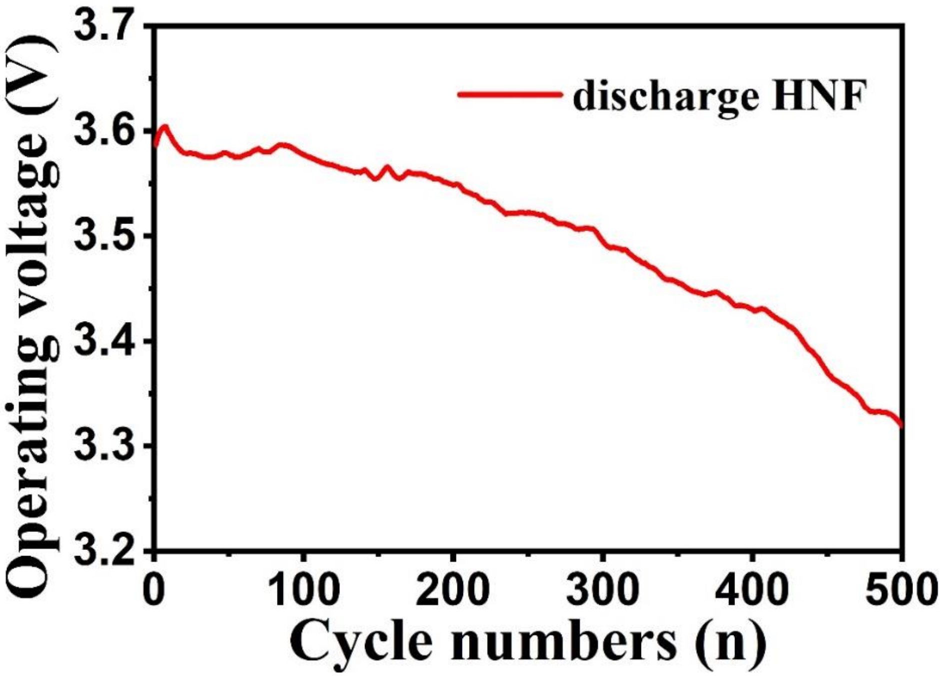

Figure S11. Correlation between discharge voltage and cycle number for HNF at the current of 1 A/g.

Table S1. Contribution of different lithium ion insertion process to theoretical specific capacity of nanowire (NW), bundle (BU) and hierarchical nanoflower (HNF) prepared with phosphoric acid.

Sample	1st Li(mAh/g)	2nd Li(mAh/g)	3rd Li(mAh/g)
HNF	167	344	436.23
BU	147	294	388.25
NW	97	244	335.12

Table S2. The performance comparison of the previous reported vanadium oxide based Li-ion battery cathodes.

Samples	Morphologies	Current density (A/g)	Max. Specific capacity (mAh/g)	References
$H_{0.39} V_2O_5$	Nanoflowers	0.1	436.23	This work
$H_2V_3O_8$/rGO	Nanobelts	0.1	377	S1
V_2O_5	Yolk-shell microspheres	0.05	317	S2
$Li_{0.0625}V_2O_5$	Nanobelts	0.03	213	S3
V_2O_5	Microspheres	0.05	286	S4
C/V_2O_5	Nanofibers	0.01	294	S5
V_2O_5/Polymer/Graphite	Nanobelts	0.03	297	S6
V_2O_5	Yolk-shell	0.3	294	S7
V_2O_5	Sisal-like	0.03	297	S8
V_2O_5/rGO	Nanowires	0.12	147	S9
V_2O_5	Nanobelts	0.3	240	S10
V_2O_5	Nanoparticles	0.3	276	S11
V_2O_5	Nanosheet-flowers	0.03	300	S12
V_2O_5	3D hollow-porous	0.1	283	S13
V_2O_5	Hierarchical spheres	0.06	275	S14

Table S3. Fitting parameters of nanowire (NW), bundle (BU) and hierarchical nanoflower (HNF) prepared with phosphoric acid.

Sample	Rs (Ω)	Rct (Ω)	Rt (Ω)	W (Ω)
HNF	5.59	205.2	55.42	0.027
BU	8.893	312.1	70.95	0.0008572
NW	2.98	404.5	118.6	0.001176

References:

[S1] Z. Liu, R. Xu, W. Wei, P. Jing, X. Li, Q. Zhu, H. Sun, Y. Dong, G. S. Zakharova, *Solid State Ionics* **2019**, 329, 74.

[S2] Y. Dou, X. Liang, G. Gao, G. Wu, *J. Alloys Compd.* **2018**, 735, 109.

[S3] W. Zhong, J. Huang, S. Liang, J. Liu, Y. Li, G. Cai, Y. Jiang, J. Liu, *ACS Energy Lett.* **2019**, 5, 31.

[S4] S. Ki Park, P. Nakhanivej, J. Seok Yeon, K. Ho Shin, W. M. Dose, M. De Volder, J. Bae Lee, H. Jin Kim, H. S. Park, *J. Energy Chem.* **2021**, 55, 108.

[S5] G.-H. An, D.-Y. Lee, H.-J. Ahn, *ACS Appl. Mater. Interfaces* **2016**, 8, 19466.

[S6] D. Chao, X. Xia, J. Liu, Z. Fan, C. F. Ng, J. Lin, H. Zhang, Z. X. Shen, H. J. Fan, *Adv. Mater.* **2014**, 26, 5794.

[S7] Y. Ma, A. Huang, H. Zhou, S. Ji, S. Zhang, R. Li, H. Yao, X. Cao, P. Jin, *J. Mater. Chem. A* **2017**, 5, 6522.

[S8] N. Wu, W. Du, G. Liu, Z. Zhou, H.-R. Fu, Q. Tang, X. Liu, Y.-B. He, *ACS Appl. Mater. Interfaces* **2017**, 9, 43681.

[S9] Y. Zhang, J. Lai, Y. Gong, Y. Hu, J. Liu, C. Sun, Z. L. Wang, *ACS Appl. Mater. Interfaces* **2016**, 8, 34309.

[S10] Y. Rong, Y. Cao, N. Guo, Y. Li, W. Jia, D. Jia, *Electrochim. Acta* **2016**, 222, 1691.

[S11] T. Kim, J. Shin, T.-S. You, H. Lee, J. Kim, *Electrochim. Acta* **2015**, 164, 227.

[S12] G. Li, Y. Qiu, Y. Hou, H. Li, L. Zhou, H. Deng, Y. Zhang, *J. Mater. Chem. A* **2015**, 3, 1103.

[S13] L. Mai, Q. An, Q. Wei, J. Fei, P. Zhang, X. Xu, Y. Zhao, M. Yan, W. Wen, L. Xu, *Small* **2014**, 10, 3032.

[S14] H. Bai, Z. Liu, D. D. Sun, S. H. Chan, *Energy* **2014**, 76, 607.
